# Development and testing of species-specific ELISA assays to measure IFN-γ and TNF-α in bottlenose dolphins (*Tursiops truncatus*)

**DOI:** 10.1371/journal.pone.0190786

**Published:** 2018-01-05

**Authors:** Kirsten C. Eberle, Stephanie K. Venn-Watson, Eric D. Jensen, Joanna LaBresh, Yvonne Sullivan, Laura Kakach, Randy E. Sacco

**Affiliations:** 1 Ruminant Diseases and Immunology Research Unit, National Animal Disease Center, Agricultural Research Service, United States Department of Agriculture, Ames, IA, United States of America; 2 Molecular Cellular and Developmental Biology Graduate Program, Iowa State University, Ames, IA, United States of America; 3 Immunobiology Graduate Program, Iowa State University, Ames, IA, United States of America; 4 Translational Medicine & Research Program, National Marine Mammal Foundation, San Diego, CA, United States of America; 5 US Navy Marine Mammal Program, San Diego, CA, United States of America; 6 Kingfisher Biotech, Inc., St. Paul, MN, United States of America; The Ohio State University, UNITED STATES

## Abstract

Monitoring the immune status of cetaceans is important for a variety of health conditions. Assays to quantify cytokines, especially pro-inflammatory cytokines, could be employed, in addition to currently available diagnostic assays, to screen for alterations in the health status of an animal. Though a number of immunological assays are readily available for humans and mice, specific assays for many veterinary species, including cetaceans such as bottlenose dolphins (*Tursiops truncatus)*, are more limited. Herein, we describe the development of IFN-gamma (IFN-γ) and TNF-alpha (TNF-α) enzyme-linked immunosorbent assays (ELISAs) specific to bottlenose dolphins. Utilizing these assays, we monitored the immune status of bottlenose dolphins from a managed population over a period of eleven months. The ELISA assays developed for bottlenose dolphins were used to measure IFN-γ and TNF-α in serum or in culture supernatants from peripheral blood mononuclear cells (PBMCs) stimulated with varying concentrations of mitogens concanavalin A (ConA) or phytohemagglutinin (PHA). Induction of TNF-α in PBMC cultures was consistently highest with 1 μg/mL ConA, while 1 μg/mL PHA induced the highest secretion of IFN-γ. Serum levels of TNF-α and IFN-γ remained relatively constant for each animal over the time period examined. CBC and plasma chemistry variables measured concurrently in the bottlenose dolphins were then examined as independent predictors of cytokine levels. We found these clinical variables were more likely to predict linear changes in serum IFN-γ and TNF-α levels compared to concentrations of these cytokines in mitogen-stimulated PBMC culture supernatants. Cytokine assays developed will be of substantial benefit in monitoring bottlenose dolphin health as an adjunct to currently available diagnostic tests.

## Introduction

While much is known regarding the immune system of humans and mice, less is known about the immune system of many veterinary species. This is especially evident for marine mammals, such as cetaceans. The U.S. Navy Marine Mammal Program (MMP) is specifically interested in improved methods to monitor the health of their bottlenose dolphins (*Tursiops truncatus)*, a managed population that has been well studied. An increase in the number of geriatric dolphins in this managed population, as well as other managed populations, has led to increased incidence of diseases associated with aging including hyperinsulinemia, metabolic syndrome, and autoimmune disease [1]. Additionally, a variety of bacterial and viral infections can infect bottlenose dolphins [2–4]. Modulation of immune function is thought to play a role in the increasing incidence of infectious diseases in marine mammals.

Increased production of a variety of pro-inflammatory cytokines is the hallmark for many diseases. Using real-time qPCR and fluorescent IHC, Eberle et al. showed that a dolphin with hyperinsulinemia, hyperglycemia, chronic inflammation, and fatty liver disease displayed a similar pro-inflammatory state as humans with type 2 diabetes and hyperinsulinemia [5]. Tumor necrosis factor-alpha (TNF-α) and interferon-gamma (IFN-γ) are two cytokines specifically useful to measure the inflammatory state of an animal [6,7]. TNF-α is primarily produced by macrophages in response to both acute and chronic conditions (trauma, sepsis, infection, rheumatoid arthritis, inflammatory bowel disease, bronchitis, and glomerulonephritis) [8, 9]. The type II IFN, IFN-γ, is involved in an array of functions in an immune response. IFN-γ produced by professional APCs can upregulate antigen processing and presentation by MHC I and II, induce leukocyte attraction, enhance natural killer cell activity, and regulate immunoglobulin production and class switching in B cells [10]. In an adaptive immune response, T lymphocytes are the key producers of IFN-γ to drive a Th1 phenotype [10].

Cytokines are critically involved in cellular communication and the measurement of these proteins would provide a functional indicator of the status of the host immune system. However, such characterization of the immune system of marine mammals has been hampered by the lack of reagents for such studies in these animals. In collaboration with Kingfisher Biotech, Inc. and the National Marine Mammal Foundation, ELISA assays specific for bottlenose dolphin have been developed. Here we show that TNF-α and IFN-γ protein can be detected by the developed ELISAs in both dolphin serum and peripheral blood mononuclear cell (PBMC) supernatant. We then determined whether CBC and plasma chemistry variables measured concurrently were predictive of cytokine levels in bottlenose dolphins.

## Materials and methods

### Study subjects

The MMP houses and cares for a population of dolphins in San Diego Bay, CA. The MMP is AAALAC-accredited and adheres to the national standards of the United States Public Health Service Policy on the Humane Care and Use of Laboratory Animals and the Animal Welfare Act. The MMP’s animal care and use program is routinely reviewed by an institutional animal care and use committee and the Department of Defense Bureau of Medicine. The animal use and care protocol for MMP dolphins in support of this study was approved by the MMP’s Institutional Animal Care and Use Committee and the Navy’s Bureau of Medicine (IACUC, Approval No. 106–2013, BUMED NRD-964). Table 1 details information about each dolphin monitored through this study including age, gender, medications, health conditions, and abnormal white blood cell count (WBC). Month 1 corresponds to June 2014.

**Table 1:** Bottlenose dolphin (*Tursiops truncatus*) clinical information.

Animal	Age (years) at onset of study	Sex	Mo of high WBC count (>10.2 x3/μL)	Mo of abnormal ESR60^1^ (>19 mm/h)	Acute Conditions (month)	Chronic Conditions	Medication (month)
**A**	35.8	M	1, 5, 7, 9	1	Pneumonia (5, 6, 7)	Dental associated osteomyelitis	Multiple antibiotics/antifungals (duration of study)
**B^2^**	10.1	M	Normal	Normal	Inflammatory hemogram—unknown etiology (3, 4, and 5)		Oral meloxicam (duration of study)
**C**	13	F	Normal	Normal	Pregnant (6 onward)		Folic acid (duration of study)
**D^2^**	44	M	3	8, 9, 10		Dental associated osteomyelitis	Oral ondansetron (11)

^1^ ESR60 = 60 min erythrocyte sedimentation rate.

^2^ Males which exhibited clinical signs of being reproductively active during this study. Animal B was noted to be reproductively active in months 3–5 of this study and animal D to be reproductively active months 1–11.

### Serum collection and PBMC isolation

Serum samples and PBMCs were collected between month 1 (June 2014) and month 11 (April 2015). For serum collection, whole blood was collected in serum separator tubes (SST). Tubes were spun at 3000 rpm for 10 min at room temperature. Serum was collected and placed at -80°C for future use.

For PBMC isolation, blood samples were collected in BD Vacutainer CPT Mononuclear Cell Preparation Tube-Sodium Citrate (BD Biosciences, 362761). Tubes were spun at 1500 x g at room temperature for 30 min. Samples were then shipped at 4°C overnight. Upon arrival, the plasma layer was removed from the top of the gel plug and diluted with an equal volume of DPBS. The sample was spun at 1200 rpm for 10 min. The supernatant was removed and RBCs were lysed. After RBC lysis and a wash with PBS, the final cell pellet was resuspended in cDMEM (Gibco, 11995–065) with 10% FBS, L-glutamine (Gibco, 25030–081), Antibiotic-Antimycotic Solution (Sigma, A5955), and MEM NEAA (Gibco, 11140–050).

### PBMC stimulation and sample isolation

The PBMC cell count was adjusted to 2x10^6^ cells/mL and 100 μL was added to a 96 well U-bottom plate. PBMCs were either stimulated with 1, 5, or 10 μg/mL of Phytohemagglutinin (PHA) Lectin from *Phaseolus vulgaris* (red kidney bean) (Sigma, L1668) or 1 μg/mL of Concanavalin A from *Canavalia ensiformis* (Jack bean) (ConA) (Sigma, L7647) or not stimulated (mock) in culture media for 48 h. ConA and PHA concentrations were based on previous studies examining mitogen stimulation of PBMCs from marine mammals [11, 12]. After 48 h, plates were spun at 1000 rpm for 5 min to pellet PBMCs. The supernatant was collected and placed at -80°C for future use.

### ELISA assays

ELISA assays were run using the bottlenose dolphin TNF-α (DIY0693P-003) or IFN-γ (DIY0690P-003) ELISA kits from Kingfisher Biotech, Inc. We optimized the protocol for each ELISA (S1 Table). Plates were coated overnight at room temperature with capture antibody in 0.05 M carbonate-bicarbonate buffer pH 9.6 (Sigma C-3041). NUNC MaxiSorp (Thermo Scientific, 439454) or Immulon 2 HB 96 well flat bottom plates were used for coating with TNF-α or IFN-γ polyclonal antibody, respectively. Plates were blocked for 1 h at room temperature with 200 uL of filtered 4% BSA in DPBS. For both ELISA assays, recombinant dolphin IFN-γ and TNF-α standards were run with 1:2 serial dilutions. The limit of detection for the IFN-γ and TNF-α ELISAs was determined to be 0.156 ng/ml. Streptavidin-HRP (R&D Systems, DY998) antibody was used and ELISA plates were developed with SureBlue TMB Microwell Peroxidase Substrate (KPL, 52-00-00). TMB Stop Solution (KPL, 50-85-05) was added to halt the reaction. Dilutions for standard, detection antibody, and streptavidin-HRP were in filtered 4% BSA in DPBS. All washes were done using a BioTek ELx405 plate washer with 0.05% Tween-20 in DPBS. The absorbance at 450 nm was measured on a Molecular Devices FlexStation 3 Reader. Representative standard curves for IFN-γ and TNF-α ELISAs are shown in S1 Fig, respectively.

### CBC and plasma chemistries

The following CBC and plasma chemistry variables (n = 48) were measured, on each bottlenose dolphin at each time point of the study, as part of routine care at the MMP using methods previously described [1]: Total white blood cell (WBC) count, red blood cell (RBC) count, hemoglobin, hematocrit, packed cell volume (PCV), mean corpuscular volume (MCV), mean corpuscular hemoglobin (MCH), mean corpuscular hemoglobin concentration (MCHC), RBC distribution width, nucleated red blood cells, platelets, mean platelet volume (MPV), neutrophils (% and total), lymphocytes (% and total), monocytes (% and total), eosinophils (% and total), glucose, blood urea nitrogen (BUN), BUN-to-creatinine ratio, uric acid, sodium, potassium, chloride, carbon dioxide, protein, albumin, calcium, inorganic phosphate, alkaline phosphatase, lactate dehydrogenase, aspartate transaminase (AST), alanine transaminase (ALT), gamma-glutamyl transpeptidase (GGT), bilirubin, total cholesterol, high density lipoprotein (HDL) cholesterol, very low density lipoprotein (VLDL) cholesterol, triglycerides, iron, creatine phosphokinase (CPK), erythrocyte sedimentation rate (ESR), estimated glomerular filtration rate (GFR), insulin, and ferritin.

### Statistical analyses

Serum IFN-γ and TNF-α levels were used in the statistical models, as well as IFN-γ produced by stimulating PBMCs with 1 μg/ml PHA for 48h and TNF-α produced by stimulating PBMCs with 1 μg/ml Con A for 48h. All 48 CBC and plasma chemistry variables measured on each animal were included in a stepwise regression model to determine which were independent predictors (P ≤ 0.05) of each of the four cytokine measurements. Independent predictors were then assessed for linear relationships with cytokine measurements to identify CBC and plasma chemistry variables that were independent, linear predictors of IFN-γ and TNF-α levels in the study dolphins.

## Results

### TNF-α and IFN-γ ELISA assays using *Tursiops truncatus* supernatants from ConA and PHA stimulated PBMCs

**Fig 1:**
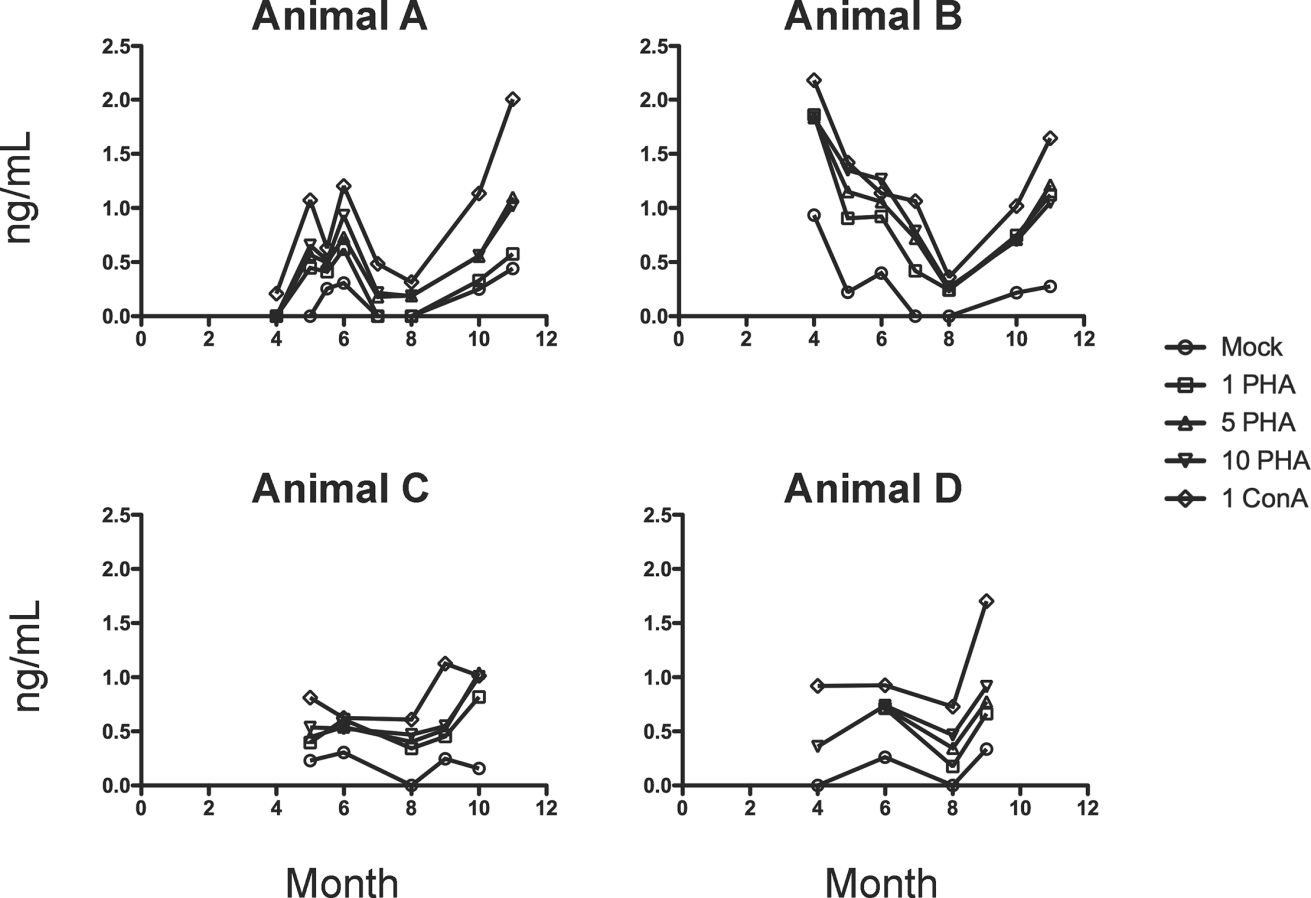
Time course of TNF-α levels (ng/mL) in supernatants from ConA or PHA stimulated PBMCs isolated from bottlenose dolphins (*Tursiops truncatus*). PBMCs isolated from *Tursiops truncatus* were stimulated with 1, 5, or 10 μg/mL PHA or 1 μg/mL ConA for 48 h in culture. Supernatants were collected and run on an ELISA assay specific for bottlenose dolphin TNF-α as described in Materials and Methods. Samples were collected monthly over an 11-month period from four bottlenose dolphins (Animals A, B, C, and D). Month of the study is denoted on the x-axis and month 1 corresponds to June 2014.

**Fig 2:**
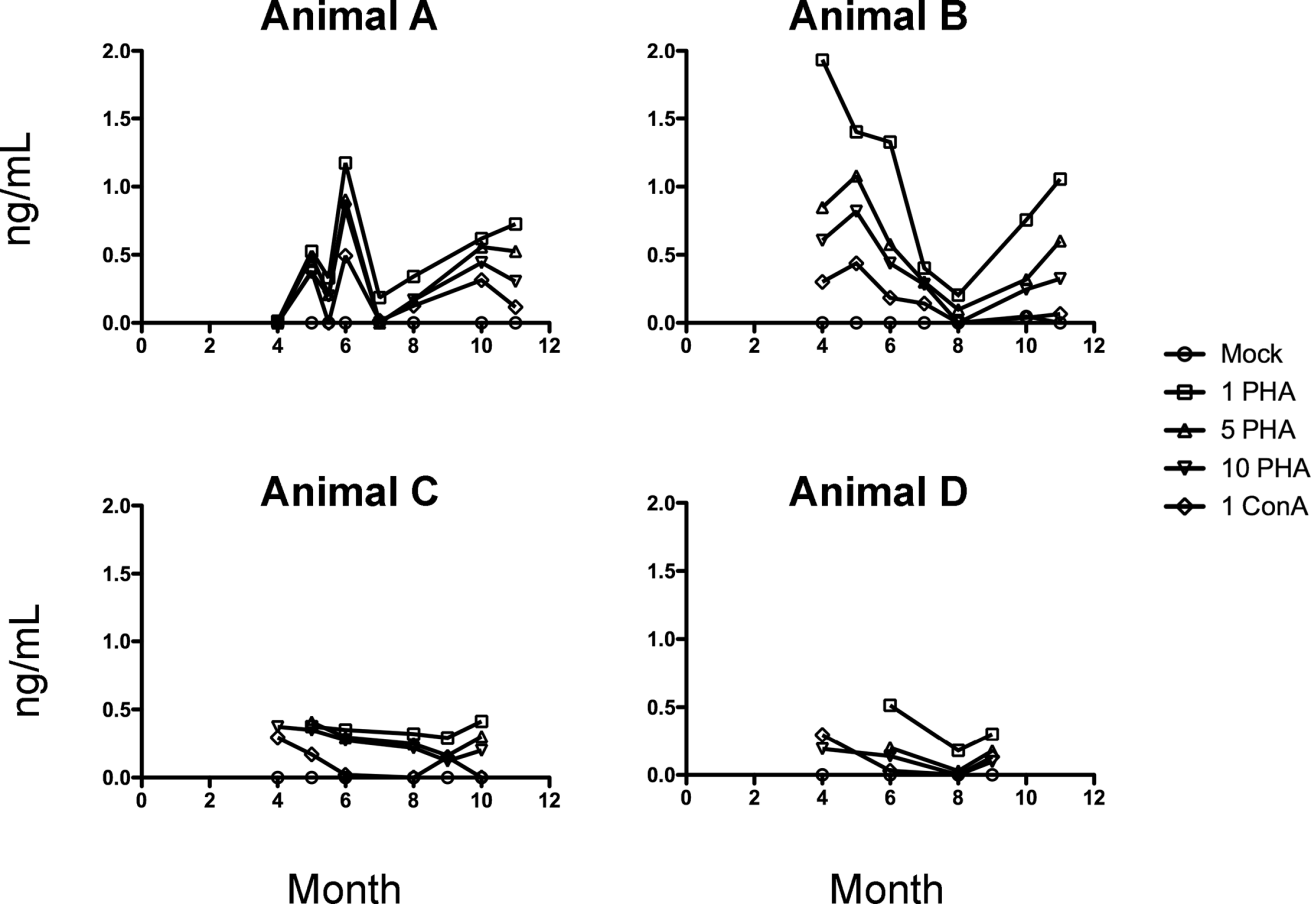
Time course of IFN-γ (ng/mL) levels in supernatants from ConA or PHA stimulated PBMCs isolated from bottlenose dophins (*Tursiops truncatus*). PBMCs isolated from *Tursiops truncatus* were stimulated with 1, 5, or 10 μg/mL PHA or 1 μg/mL ConA for 48 hours in culture. Supernatants were collected and run on an ELISA assay specific for bottlenose dolphin IFN-γ as described in Materials and Methods. Samples were collected monthly over an 11-month period from four bottlenose dolphins (Animals A, B, C, and D). Month of the study is denoted on the x-axis and month 1 corresponds to June 2014.

As an adjunct to diagnostic assays to monitor bottlenose dolphin health, we sought to develop a set of data on pro-inflammatory cytokine levels through the establishment of specific cytokine/chemokine ELISAs. With this in mind, protein levels of TNF-α (Fig 1) and IFN-γ (Fig 2) in supernatants of PBMCs stimulated with ConA and PHA were measured by a bottlenose dolphin specific ELISA. In this study, PBMCs were collected monthly over a period of 11 months; however, samples were not able to be collected for each animal every month.

As can be seen in Figs 1 and 2, there was overall high variability of the TNF-α and IFN-γ levels. The variability in TNF-α and IFN-γ levels over time may be due to changes in diet, breeding patterns, or medication received. Levels of IFN-γ in Animals C and D were low and seemed to stay consistent through the months analyzed. Interestingly, Animal A was diagnosed with pneumonia at months 5, 6, and 7 of the study. This is where noticeable instability of both TNF-α and IFN-γ occurred (month 5, 5.5, and 6). Animal C became pregnant at month 6, but no noticeable differences in cytokine secretion were observed upon pregnancy.

Overall, 48 h PBMC stimulation with 1 μg/mL ConA induced the highest production of TNF-α, while 1 μg/mL PHA stimulated the uppermost levels of IFN-γ in all animals. We have limited data (not shown) where PBMCs were stimulated for 24 h. Based on the few samples collected, it appears that 24 h stimulation was not optimal to induce sufficient cytokine production for analysis. We therefore recommend a 48 h induction as shown in Figs 1 and 2.

### Time course of TNF-α and IFN-γ levels in *Tursiops truncatus* serum samples

**Fig 3:**
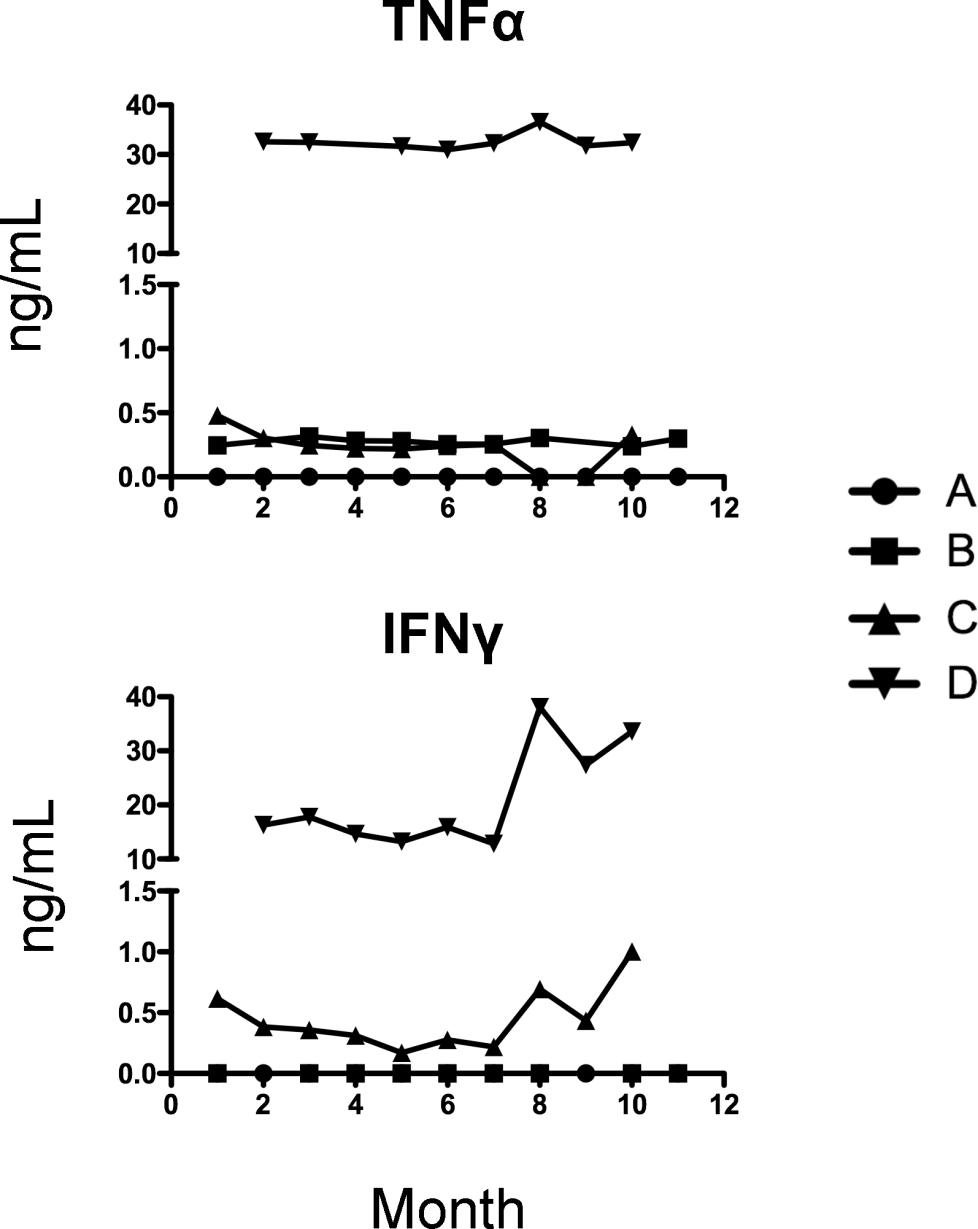
Time course of TNF-α and IFN-γ levels (ng/mL) in bottlenose dolphin (*Tursiops truncatus*) serum samples. A serum sample from four bottlenose dolphins (Animals A, B, C, and D) was collected monthly over an 11-month period. Samples were run on ELISA assays specific for bottlenose dolphin TNF-α and IFN-γ as described in Materials and Methods. Month of the study is denoted on the x-axis and month 1 corresponds to June 2014.

Shown in Fig 3 are serum levels of TNF-α and IFN-γ that were measured by dolphin specific ELISA assays. Serum levels of TNF-α and IFN-γ remained relatively constant as compared to the levels in supernatants from stimulated PBMC cells shown in Figs 1 and 2, which fluctuated over time. Importantly, levels of TNF-α and IFN-γ were consistent on a per animal basis. That is, Animal D was seen to have higher TNF-α and IFN-γ levels and those levels remained elevated throughout the study. The possible acute infection in Animal A between 5 and 7 months, did not result in significant changes in cytokine levels observed in serum samples. However, changes in serum levels of TNF-α and IFN-γ may be transient during an acute infection and we simply missed the window of time to measure any fluctuations in TNF-α or IFN-γ from baseline levels in this animal.

### CBC and plasma chemistries as linear predictors of cytokines

CBC and plasma chemistry variables were more likely to predict linear changes in serum IFN-γ and TNF-α levels compared to PBMC-stimulated IFN-γ and TNF-α levels (Table 2). Higher RBC distribution width, insulin, protein, and creatinine; and lower estimated glomerular filtration rate (eGFR) predicted higher serum IFN-γ. Higher RBC distribution width, insulin, cholesterol, hematocrit, creatinine and ESR predicted higher serum TNF-α.

**Table 2:** Summary of blood-based health indices that independently predicted a linear change in IFN-γ and TNF-α levels in bottlenose dolphins (*Tursiops truncatus*).

Cytokine	Blood-based Index	Stepwise Regression P value	Simple Linear Regression R^2^ value
IFN**-γ** (serum)	RBC distribution width	< 0.0001	(+) 0.47
	Insulin	< 0.0001	(+) 0.42
	Protein	0.0009	(+) 0.40
	Creatinine	0.006	(+) 0.35
	eGFR	< 0.0001	(-) 0.24
	GGT	0.0002	-
	Iron	0.04	-
	MCH	0.01	-
	Glucose	0.04	-
TNF**-α** (serum)	Insulin	< 0.0001	(+) 0.63
	RBC distribution width	< 0.0001	(+) 0.55
	Cholesterol	0.001	(+) 0.44
	HCT	0.0003	(+) 0.33
	Creatinine	0.01	(+) 0.29
	ESR	0.0005	(+) 0.19
	GGT	0.03	-
	ALT	0.01	-
	Inorganic phosphate	0.006	-
	Nucleated RBCs	0.04	-
	Calcium	0.03	-
IFN**-γ** (PBMCs stimulated with 1 μg/ml PHA for 48h)	Alkaline phosphatase	0.0006	(+) 0.50
	CPK	0.03	(+) 0.47
	MCH	0.05	-
	HGB	0.04	-
	Eosinophils (AC)	0.02	-
TNF**-α** (PBMCs stimulated with 1 μg/ml Con A for 48h)	GFR	0.02	(-) 0.29
	ESR	0.003	-
	Protein	0.03	-
	Platelets	0.008	-
	Lymphocytes (AC)	0.009	-
	Neutrophils (%)	0.01	-
	MCV	0.009	-
	BUN:creatinine	0.05	-
	Chloride	0.005	-

## Discussion

Cytokines play an important role in inflammatory processes, in the production of antibodies, and in cell mediated-immune responses. Thus, cytokine protein levels could be used in identifying modifications or alterations in the cetacean immune system as a result of environmental insults and serve as tools in characterizing immune responses to pathogens and/or vaccines. Here we developed and tested two bottlenose dolphin specific ELISA assays: TNF-α and IFN-γ. Each animal monitored herein exhibited a distinct cytokine pattern over the 11 months in both serum samples and in stimulated PBMC supernatants. To determine any deviation in health status for a specific animal, that individual’s baseline cytokine level under normal conditions would need to be determined. Overall, serum samples may be best to use for monitoring disease state over time. In addition to easier collection and storage, serum levels of TNF-α and IFN-γ seemed to remain steady over time. It would therefore be easier to observe a change in cytokine levels if an illness or other changes in health condition did occur.

We and others have used cross-reactive antibodies specific for human, mouse, porcine, and bovine on cetacean samples [5, 13, 14]. However, cross-species reactivity of available Ab to cytokines or chemokines cannot be universally expected for all cytokines. Furthermore, specific amino acid substitutions in a functional epitope recognized by an antibody could cause considerable reductions in relative binding affinity of the antibody paratope. It is known that only a subset of contact residues, typically between three and five within an epitope, contribute significantly to the binding energy [15]. These contact residues are commonly referred to as hot spot residues, which together form a functional epitope [16]. Therefore, between species differences in these contact residues could result in reductions in antibody binding affinity that would likely impact relative cytokine quantification by standard curve. Though the development of recombinant cytokines and appropriate antibodies is costly and time consuming, the use of species-specific reagents has several advantages. In addition to more specific, higher affinity antibody binding, lower background levels of detection should be possible with species-specific antibodies. In a sandwich ELISA, using the same antibody for coating and detection is not optimal. Pairing a monoclonal antibody (mAb) with a polyclonal antibody can enhance signal and increase the chances of capturing the antigen of interest from a complex sample.

In collaboration with Washington State University Monoclonal Antibody Center, mAb production for specific cytokines has been initiated. These mAb’s will be utilized in sandwich ELISAs to further enhance the specificity and sensitivity of bottlenose dolphin-specific assays. We are continuing to develop dolphin-specific cytokine and chemokine ELISAs. Since the initiation of the present study, additional dolphin specific ELISA assays have become available: IL-1 receptor antagonist, IL-2, IL-4, IL-8, and IL-17A. The TNF-α and IFN-γ ELISA assays described herein and others that are available from Kingfisher Biotech, Inc. will be useful in moving forward with health monitoring and disease detection in captive, managed, and free-ranging bottlenose dolphins.

In this study, to assess potential clinical relevance of IFN-γ and TNF-α levels in dolphins, elevated insulin was an independent predictor of elevated serum levels of cytokines IFN-γ and TNF-α in four dolphins monitored over 10 months. Elevated cholesterol also predicted higher serum TNF-α. There were no independent, linear associations identified between white blood cell counts and IFN-γ or TNF-α, suggesting that these cytokines were better indicators of subclinical, chronic changes than acute infections. Similar to humans, dolphins can develop insulin resistance with hyperinsulinemia and dyslipidemia with hypercholesterolemia as part of a cluster of conditions called metabolic syndrome [17]. Chronic systemic inflammation, including elevated circulating cytokines such as TNF-α, has been previously associated with insulin resistance and hypercholesterolemia in mammals [18, 19]. The positive associations between increasing IFN-γ or TNF-α and insulin, as well as TNF-α with cholesterol, in this study support a similar role of inflammation and elevated cytokines in metabolic syndrome in dolphins.

## Conclusions

In conclusion, we have developed ELISA assays specific for bottlenose dolphin cytokines. We have shown that TNF-α and IFN-γ protein can be detected in the developed ELISAs using either bottlenose dolphin sera or supernatants from mitogen-stimulated PBMCs. Longitudinal analyses indicated that each dolphin exhibited a distinct cytokine pattern over the 11 month period, with serum levels of TNF-α and IFN-γ remaining relatively level over time. Finally, we observed positive associations between increasing IFN-γ or TNF-α and insulin, as well as TNF-α with cholesterol, supporting similar roles of inflammation and elevated cytokines in bottlenose dolphins to that described for human patients. Cytokine ELISAs will be of substantial benefit as an adjunct to currently available diagnostic tests in monitoring bottlenose dolphin health.

## Supporting information

S1 Table Protocols for bottlenose dolphin TNF-α and IFN-γ specific ELISA assays. (DOCX)

S1 Fig Representative standard curves for bottlenose dolphin (*Tursiops truncatus*) IFN- γ and TNF-α ELISA assays. (TIF)
