# Supplementary material for: Development and testing of species-specific ELISA assays to measure IFN-γ and TNF-α in bottlenose dolphins (Tursiops truncatus)
Source: PLoS One. 2018 Jan 5;13(1):e0190786. doi: 10.1371/journal.pone.0190786 (PMC5755893; doi:10.1371/journal.pone.0190786)
Supplement: S1 Table — (DOCX) [file pone.0190786.s001.docx]

| **Step** | | **TNF**-α **ELISA** | | **IFN**-γ **ELISA** | |
| --- | --- | --- | --- | --- | --- |
|  |  | **Amount** | **Duration** | **Amount** | **Duration** |
| **1** | **Coating** | 2 µg/mL, 100 µL | Overnight, RT | 1 µg/mL, 100 µL | Overnight, RT |
| **2** | **Blocking** | 4% BSA in PBS, 100 µL | 1 hour, RT | 4% BSA in PBS, 100 µL | 1 hour, RT |
| **3** | **Highest standard** | 10 ng/mL, 50 µL | 1 hour, RT | 40 ng/mL, 50µL | 2 hours, 37^o^C |
|  | **Lowest standard** | 0.156 ng/mL, 50 µL |  | 0.156 ng/mL, 50µL |  |
|  | **Sample^1^** | 50 µL |  | 50 µL |  |
| **4** | **Biotinylated secondary antibody** | 0.1 µg/mL, 100 µL | 1 hour, RT | 1 µg/mL, 100 µL | 1 hour, RT |
| **5** | **Streptavidin-HRP** | 1:100, 100 µL | 30 min, RT | 1:100, 100 µL | 30 min, RT |
| **6** | **Develop with TMB** | 100 µL | 30 min, RT | 100 µL | 10 min, RT |
| **7** | **TMB stop solution** | 100 µL |  | 100 µL |  |

**S1 Table. Protocols for bottlenose dolphin TNF**-α **and IFN**-γ **specific ELISA assays.**

^1^ Optimal sample dilutions should be determined within each laboratory.
