# Supplementary figures and images for: Development and testing of species-specific ELISA assays to measure IFN-γ and TNF-α in bottlenose dolphins (Tursiops truncatus)
Source: PLoS One. 2018 Jan 5;13(1):e0190786. doi: 10.1371/journal.pone.0190786 (PMC5755893; doi:10.1371/journal.pone.0190786)

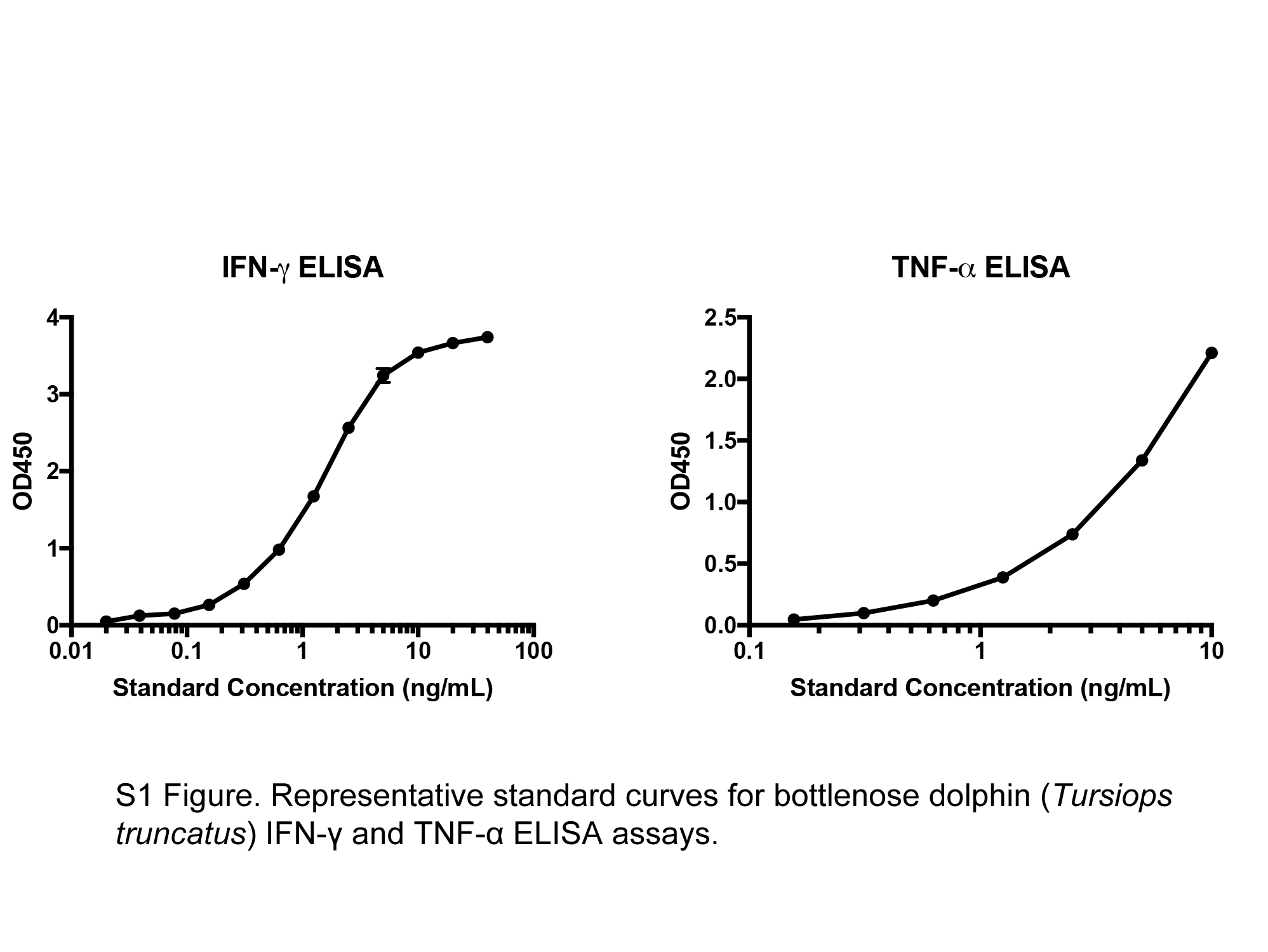

Supplement: S1 Fig — (TIF) [file pone.0190786.s002.tif]
